# Inhibition of Urinary Macromolecule Heparin on Aggregation of Nano-COM and Nano-COD Crystals

**DOI:** 10.3390/molecules20011626

**Published:** 2015-01-19

**Authors:** Yan Ou, Jun-Fa Xue, Cai-Yan Tan, Bao-Song Gui, Xin-Yuan Sun, Jian-Ming Ouyang

**Affiliations:** 1 Department of Nephrology, the Second Hospital of Xi’an Jiaotong University, Xi’an 710004, China; 2 Institute of Biomineralization and Lithiasis Research, Jinan University, Guangzhou 510632, China

**Keywords:** nanocrystal, calcium oxalate, heparin, aggregation, XPS, TEM

## Abstract

*Purpose*: This research aims to study the influences of heparin (HP) on the aggregation of nano calcium oxalate monohydrate (COM) and nano calcium oxalate dihydrate (COD) with mean diameter of about 50 nm. *Method*: The influences of different concentrations of HP on the mean diameter and Zeta potential of nano COM and nano COD were investigated using a nanoparticle size Zeta potential analyzer. *Results*: HP could be adsorbed on the surface of nano COM and nano COD crystals, leading to an increase in the absolute value of Zeta potential on the crystals and an increase in the electrostatic repulsion force between crystals. Consequently, the aggregation of the crystals is reduced and the stability of the system is improved. The strong adsorption ability of HP was closely related to the -OSO_3_^−^ and -COO^−^ groups contained in the HP molecules. X-ray photoelectron spectroscopy confirmed the coordination of HP with Ca^2+^ ions of COM and COD crystals. *Conclusion*: HP could inhibit the aggregation of nano COM and nano COD crystals and increase their stability in aqueous solution, which is conducive in inhibiting the formation of calcium oxalate stones.

## 1. Introduction

Calcium oxalate (CaOx) is the major inorganic component of urinary calculi. CaOx stones only contain a minimal amount of calcium phosphate, which could account for approximately 9%–16.3% [1,2,3]; calcium phosphate is usually present in the “core” of a stone [4,5].

The formation of CaOx stones is closely related to the supersaturation, nucleation, growth, aggregation, and solid phase transformation of CaOx [6]. However, no significant difference exists in the supersaturation degree of stone salts between stone-forming patients and normal persons. The inhibitors in urine play an important role in stone formation. Compared with that of stone-forming patients, the urine of normal persons has more types of inhibitors with higher concentration and stronger activity. These inhibitors include some small-molecule inorganic salts, such as citrate and pyrophosphate, and urinary macromolecules, such as glycosaminoglycan (GAGs), nephrocalcin, Tamm-Horsfall protein, and prothrombin fragment 1 [7,8].

Both growth and aggregation of crystals could increase crystal size. Crystal growth is a much slower process, so the urinary crystals were excreted from the body without growing into an appropriate size to be trapped in the duct of Bellini with an inner diameter of 7–23 microns in a transit time across the kidney of 5–10 min. However, the aggregation of urinary crystals can be finished within a short time, namely, the size of urinary crystals can increase rapidly through crystal aggregation, which is the key to the growth of urinary stones [9].

The aggregation of CaOx crystals can be caused by many factors, such as increase in the concentration of oxalate and Ca^2+^ ions in urine, increase in ionic strength, and decrease in the concentration of urine inhibitions [10].

GAGs includes eight components: heparin (HP), heparan sulfate (HS), chondroitin sulfate A (C_4_S), chondroitin sulfate C (C_6_S), dermatan sulfate (also known as chondroitin sulfate B), hyaluronic acid (HA), keratin sulfate (KS) and chondroitin (CS). All the eight components of GAGs are present in the urinary stone matrix. However, different types of GAGs are found in different kinds of stones. For example, HA is present in apatite and magnesium ammonium phosphate stones. HS has been detected in calcium oxalate monohydrate (COM) and uric acid stones, and HA and HS have been found in calcium oxalate dihydrate (COD) stone. Although C_4_S and C_6_S are the highest components of urinary GAGs, they are only found in individual apatite [11,12].

GAGs is an important urinary macromolecule that inhibits urinary stone formation [13,14,15]. The concentration of GAGs in 24 h urine of stone patients is significantly lower than that in controls, and it is higher in men than in women. The concentration of GAGs in normal people is 8.22 mg/L ± 0.60 mg/L (male) and 7.97 mg/L ± 0.43 mg/L (female), respectively, whereas that in stone patients is 2.97 mg/L ± 0.43 mg/L (male) and 2.32 mg/L ± 0.24 mg/L (female). Given the higher GAGs concentration in children’s urine, the incidence of pediatric urolithiasis is less than that in adults [16]. The deficiency of inhibitors in lithogenic urine is one of the important factors for the rapid aggregation of urinary crystals.

Many reports are available about the inhibitive effects of urinary macromolecules on CaOx formation [14,17], especially chondroitin sulfate A (C_4_S) and chondroitin sulfate C(C_6_S) [18], due to their higher content in GAGs.

Our study focuses on the influence of HP on aggregation of nano COM and nano COD crystals because of the following reasons: (i) The proportion of anion groups in the HP molecule (such as -OSO_3_^−^, -COO^−^) is the highest among the eight components of GAGs. The inhibition ability of GAGs on the nucleation, growth, and aggregation of CaOx crystals is closely related to their molecular structure, especially the anion group (such as -OSO_3_^−^ and -COO^−^) of GAGs molecule. The degree of sulfation of GAGs is a regulating factor in stone formation. The higher the degree of sulfation of GAGs, the stronger the ability to combine with free Ca^2+^, and the saturation of CaOx suspensions is decreased. Furthermore, the negative charge formed by the high degree of sulfation of GAGs could increase the Zeta potential of crystals, thereby inhibiting the growth and aggregation of CaOx crystals; (ii) A lesser number of reports are available on the inhibition of HP on nano COM and nano COD crystals. The probability of COM present in urine of stone patients is much higher than that in healthy controls, whereas that of COD is the opposite; (iii) HP is widely used as an anticoagulant in kidney dialysis and acute coronary syndromes [19,20]. It is valuable in studying *in vitro* the relationship between the use of heparin and the occurrence of renal stone.

**Figure 1:**
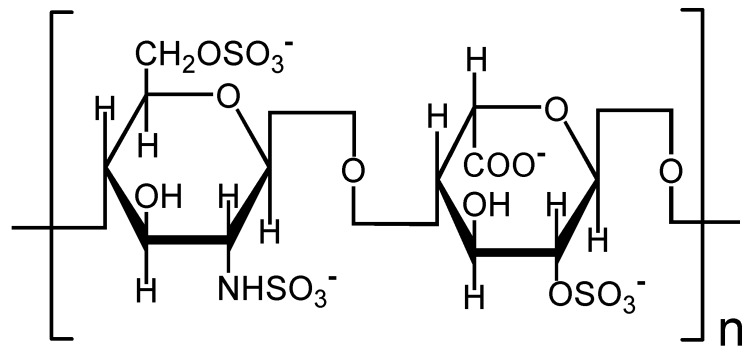
Chemical structure of the disaccharide repeating unit of heparin. (**left**): glucosamine (GlcN); (**right**): L-iduronic acid (IdoA).

HP is a straight-chain GAGs that is composed of hexuronic acid and hexosamine alternately connected by β_1-3_ and β_1-4_ bonds (Figure 1) [21]. The average molecular weight of HP is about 12,000–15,000 Da. The negative charge from the sulfate group and uronic acid in HP molecule can bind with Ca^2+^, thus decreasing the supersaturation of CaOx solution and inhibiting nucleation, growth, and aggregation of CaOx crystals. Furthermore, HP can protect the mucosa of the urinary tract, thus preventing crystal adhesion and internalization by the cell.

Other researchers hold different views about the role of HP. Kavanagh [22] once added HP into the diluted urine of stone-forming patients and controls, and found that although the nucleation rates of CaOx decreased, growth rate increased. Kok *et al.* [23] believed that high concentration of HP had no effect on agglomeration of CaOx despite the potent crystal growth inhibitory activity.

The formation of millimeter-sized stones originates from the further growth and aggregation of micron-sized crystals. The latter originates from the aggregation and growth of nano-sized crystals in urine.

Several reports [4,24] have recently shown that the presence of phosphates causes the initial formation of amorphous calcium phosphate (ACP) clusters. These ACP clusters have a crucial role in the nucleation of calcium oxalate stones by promoting the aggregation of amorphous calcium oxalate precursors at early induction times. Our previous study [6] found that nanocalcium phosphate could function as a central nidus and induce calcium oxalate stone formation.

Although phosphate has an important effect on the formation of calcium oxalate stones [25,26], the main inorganic component of renal stones is calcium oxalate, including COM and COD.

In fact, different sizes of COM and COD crystals are present in urine [6,27,28], ranging from a few nanometers to hundreds of microns. The growth and aggregation process may be different for these urinary crystals with different sizes.

This study aims to investigate the influences of the urinary inhibitor HP on aggregation of the formed calcium oxalate crystals, including nano COM and COD crystals, to elucidate the mechanism of urinary stone formation from a different perspective. This work would help in understanding the growth process of nano-sized crystals into micron-sized crystals in urine and contribute to the development of polysaccharide inhibitor drugs.

## 2. Results and Discussion

### 2.1. HP Inhibits the Aggregation of Nano COM and Nano COD Crystallites in Aqueous Solution

**Figure 2:**
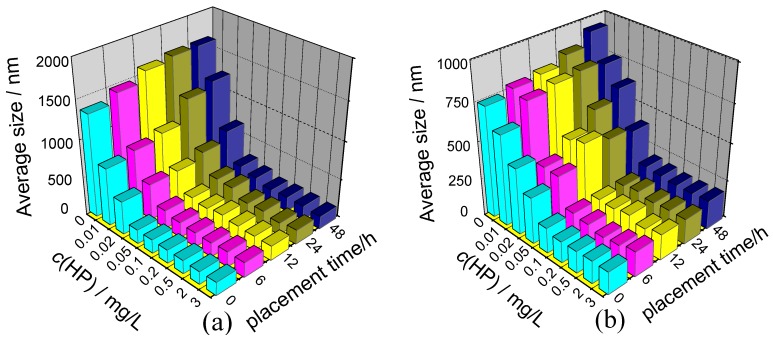
Effect of *c*(HP) and placement time (*t*) on the mean diameter of nano COM and nano COD crystals. (**a**) Nano COM; (**b**) Nano COD.

A nanoparticle size analyzer was used to study the effect of HP concentration on mean diameter of nano COM and nano COD crystals. The results are shown in Figure 2.

(1) At *c(HP)* = 0, wherein the crystals were dispersed in pure water, the mean diameters of nano COM and nano COD were 1343 and 734 nm, respectively, indicating serious aggregation of two nano crystals because the real diameters of nano COM and COD are about 50 nm.
(2) The aggregation of nano COM or nano COD was inhibited after HP was added into the solution, leading to an obvious decrease in their mean diameters. When *c(HP)* was increased to 0.10 mg/L, the mean diameter of nano COM (Figure 2a) and nano COD (Figure 2b) crystals abruptly decreased to about 200 nm, respectively.

When *c*(HP) = 0.20–3.0 mg/L, the mean diameter of nano COM or nano COD crystals barely changed and remained at around 190 and 160 nm, respectively. This result indicated that the dispersion degree of nano COM or nano COD crystals in the HP solution was the maximum at this concentration region. 

However, Figure 2 shows clear inhibitory activity on agglomeration of nano COM and nano COD crystals. The differences between our results and the findings of Kok [23] are due to the following reasons: 

First, the studied concentration range was different. The concentration range used by Kok [23] was *c*(HP) = 0.8–100 mg/L, whereas it was *c*(HP) = 0–3 mg/L in our study. Figure 2 demonstrates that the inhibition effect of HP on aggregation of CaOx crystals rapidly increased with the increase of *c*(HP) at low concentration (*c*(HP) = 0–0.10 mg/L), which made the particle size rapidly decrease. At higher concentration (*c*(HP) = 0.10–3 mg/L), the inhibition effect of HP on aggregation of CaOx crystals reached the maximum. Therefore, the particle size barely changed with the further increase of *c*(HP). The concentration of HP used by Kok [23] was too large (*c*(HP) = 0.8–100 mg/L), which was far more than the saturation concentration to reach maximum inhibition effect. In such case, obtaining the relative difference of inhibition effect of HP on CaOx crystals with the increase of *c*(HP) is difficult. Thus, Kok [23] concluded that HP had no effect on agglomeration of CaOx crystals. The inhibition effect of HP on the aggregation of CaOx crystals reached the maximum in the concentration range they used. Therefore, our result does not contradict that of Kok [23]. Meanwhile, the inhibition effect of heparin on the aggregation of CaOx crystals has been confirmed by many reports [29,30,31].

Second, Kok *et al*. [23] has already explained the difference between their results and those of other researchers. For the measurement of the crystallization processes, well-designed systems must be used. Both experimental parameters (ionic strength, pH, seed type, seed concentration) and physicochemical processes (growth, agglomeration) determine the measured growth rates and crystal size distributions.

### 2.2. HP Makes the Zeta Potentials Became Negative on the Surface of Nano COM and COD Crystallites

The magnitude of Zeta potential indicates the degree of electrostatic repulsion or attraction between charged particles, and it is one of the important basic parameters that affect the stability of particles in the solution. Zeta potential can reflect the type and amount of charges on the particle surface. The solvent, pH, ionic strength (such as NaCl concentration), particle concentration, and the properties of the particle itself greatly influence the Zeta potential, so the results are comparable only under the same conditions.

**Figure 3:**
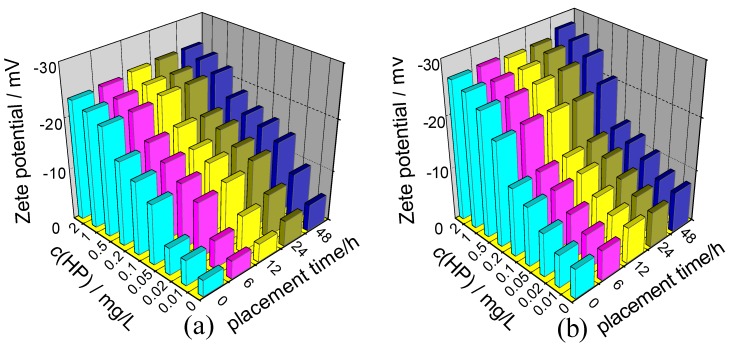
Effect of *c*(HP) and placement time (*t*) on Zeta potential of nano COM and nano COD crystals. (**a**) nano COM; (**b**) nano COD. Ion concentration: 0.15 mmol·L^−^^1^ NaCl.

Under the same conditions, the greater the absolute value of the Zeta potential of the crystals, the larger the electrostatic repulsion force between the crystals. As a result, the aggregation and deposition of the crystals become more difficult. The Zeta potentials of nano COM and COD crystals in the presence of different *c*(HP) are studied under the same condition and are shown in Figure 3, which demonstrates the following.

(1) All the Zeta potential values were negative. It did not matter how much the concentration of HP was (in the region from 0–2 mg/L), whether it was nano COM or nano COD, or whether HP was added or the solution was placed 48 h after HP was added. All the Zeta potential values were negative.

The HP molecule contained many -OSO_3_^−^ and -COO^−^ groups, both of which could form coordination bonds with Ca^2+^ ions on the calcium oxalate crystal surface (Figure 1). Furthermore, HP contained many -OH groups, which could form hydrogen bonds with Ox^2−^ ions on the crystal surface. Thus, the polyanionic HP was adsorbed on the surface of nano COM or COD crystals, leading to an increase in surface negative charges, which made their Zeta potential negative.

(2) At low concentration (*c*(HP) = 0–0.10 mg/L), the absolute value of the Zeta potential on COM and COD crystals increased rapidly with the increase of *c*(HP). However, at higher concentration (*c*(HP) = 0.20–2.0 mg/L), this increase became slower because the adsorption of HP on the crystallite surface reached saturation at this concentration region.
(3) The effect of placement time on Zeta potential was lesser. When the suspensions of nano COM and nano COD crystals were placed from 0 h to 48 h, the Zeta potential of the crystals became slightly negative because the adsorbed HP molecules on the crystallite surface became tighter and more orderly with the increase of placement time. This phenomenon led to an increase in the amount of the adsorbed HP molecules. The adsorbed amount of HP molecules increased finitely, so Zeta potential barely changed.

### 2.3. TEM Images and XRD Patterns of Nano COM and Nano COD Crystals in the Presence of HP

**Figure 4:**
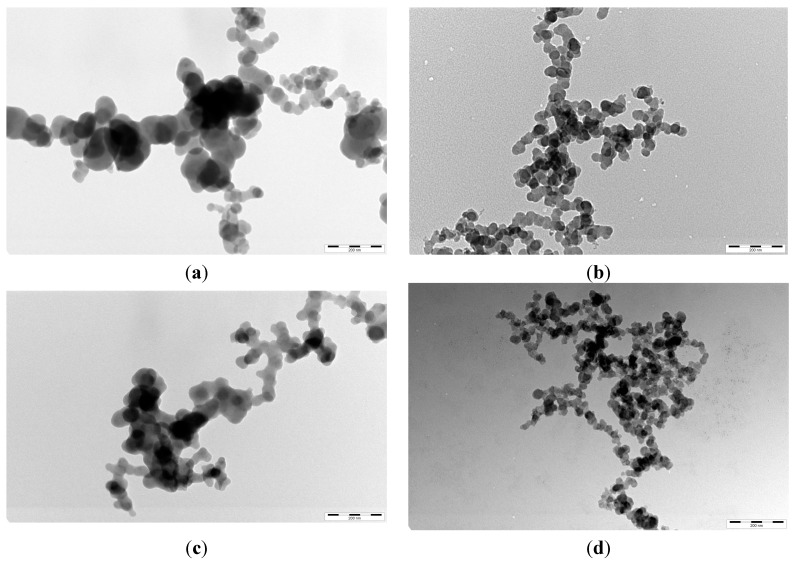
TEM images of nano COM and nano COD crystals in presence of different concentration of HP. (**a**,**b**) COM; (**c**,**d**) COD; (a,c) *c*(HP) = 0.02; (b,d) *c*(HP) = 0.20 mg/L. Incubation time: 24 h. Scale bars: 200 nm.

Figure 4 shows the TEM images of nano COM and nano COD crystals in different concentrations of HP solution after incubation for 24 h. In pure water, the aggregation degree of nano COM and nano COD crystals was high (not shown), so the mean diameter of nano COM and nano COD crystals was large. The size of crystal aggregates of nano COM or COD crystals in the presence of 0.20 mg/L HP (Figure 4b,d) was obviously less than that in presence of 0.02 mg/L of HP (Figure 4a,c). The dispersion degree of nano COM or COD crystals in the suspension increased with *c*(HP) increase. The result of TEM observation is consistent with the detection results of the nanoparticle size analyzer (Figure 2). The Zeta potential of the crystals became negative and the electrostatic repulsion between the crystals increased after HP was adsorbed on the crystal surface.

Many anionic functional groups are present in HP molecules, such as -OSO_3_^−^, -COO^−^ and -OH groups. Therefore, the density of negative charges in HP was high. These anionic groups can bind with Ca^2+^ ion through electrostatic interaction and decrease the supersaturation of CaOx solution directly, thus inhibiting the growth of CaOx crystals. The inhibition of HP on the aggregation of nano COM and nano COD was attributed to the specific adsorption of HP on the surface of COM and COD, so the Zeta potentials on the crystals were highly negative (Figure 3), leading to an increase in the electrostatic repulsion between the crystals.

Among the specific combination of HP and Ca^2+^ ion, the 2-NHSO_3_^−^ and 6-OSO_3_^−^ of glucosamine (GlcN) residue on HP and 5-COO^−^ of iduronic acid (IdoA) residue on HP were more important than others [32,33] (Figure 1). If GlcN residue lost the 6-OSO_3_^−^, the interaction of HP with Ca^2+^ ion would be weakened [34]. If GlcN residue lost the 2-NHSO_3_^−^, HP could not specifically bind with Ca^2+^ ion [32]. If 5-COO^−^ of IdoA residue is esterified or protonized, HP could not bind with Ca^2+^ as well [34]. However, the loss of 2-OSO_3_^−^ from IdoA residue almost had no effect on the specific combination of HP and Ca^2+^.

**Figure 5:**
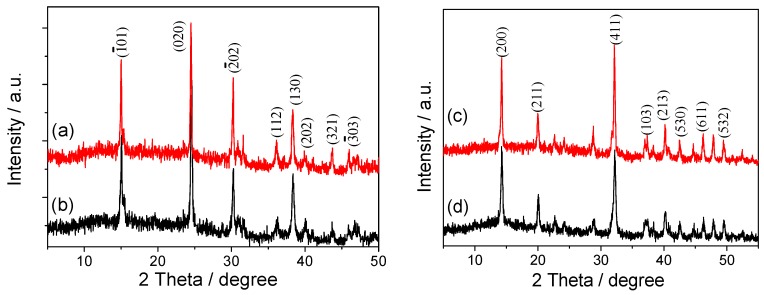
XRD patterns of the synthesized nano COM and nano COD crystals and the crystals in presence of 0.20 mg/L HP, respectively. (a,b) COM; (c,d) COD. *c*(HP): (a,c) the synthesized crystals; (b,d) the crystals in presence of 0.20 mg/L HP. Incubation time: 24 h.

Figure 5 showed the XRD patterns of the synthesized nano COM and nano COD crystals and the crystals in presence of 0.20 mg/L HP, respectively. For nano COM crystals (Figure 5a), we detected the peaks at *d* = 0.593, 0.365, 0.296, 0.249, 0.235, 0.227, 0.207 and 0.197 nm, which were assigned to (_1_01), (020), (_2_02), (112), (130), (202), (321) and (_3_03) planes of COM (PDF card number: 20-0231) [35]. For nano COD crystals (Figure 5c), we detected the peaks at *d* = 0.618, 0.442, 0.277, 0.241, 0.224, 0.212, 0.196 and 0.190 nm, which were assigned to (200), (211), (411), (103), (213), (530), (611) and (532) planes of COD (PDF card number: 20-0233). Because HP does not produce XRD peak, the XRD patterns of COM and COD crystals after combined with HP were basically the same as those before combined, respectively.

### 2.4. X-ray Photoelectron Spectroscopy (XPS) Spectra of the Interaction between HP and Crystals

**Figure 6:**
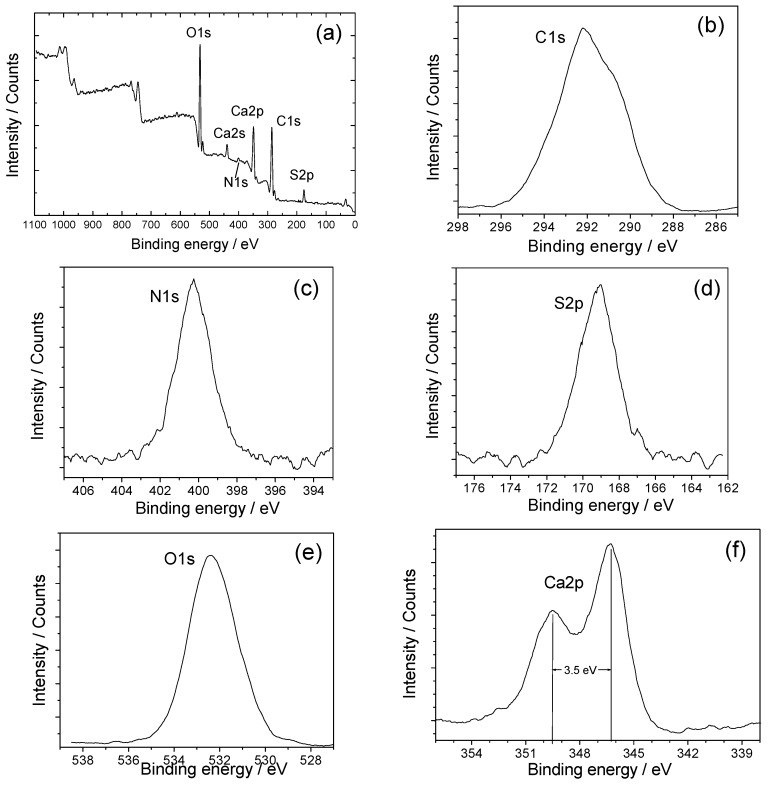
XPS survey scan for (**a**) COM-HP; and the narrow scans of (**b**) C*1s*; (**c**) N*1s*; (**d**) S*2p*; (**e**) O*1s*; and (**f**) Ca*2p*.

The types of interaction between HP and CaOx crystals were characterized by XPS spectra. The results of COM and COD were similar. Figure 6 shows the XPS survey scans of COM-HP, a compound formed by interaction between 2.0 mg/L HP and COM crystals. In addition to the constituents of HP, as well as the C*1s*, N*1s*, S*2p*, and O*1s* core level peaks, the characteristic absorption peak of Ca*2p* was also observed, which indicated the coordination of HP with COM crystals.

Figure 6b–f show the narrow scans of COM-HP in some regions. These peaks were due to C*1s*, N*1s*, S*2p*, O*1s*, and Ca*2p*, respectively. The binding energy data (E_b_) of different elements in COM, COD, HP, COM-HP, and COD-HP are shown in Table 1.

After the correction of charge transfer with C*1s* = 285.00, the appearance of a low-energy component with E_b_ = 399.5 eV in the N*1s* band spectra in the XPS spectra of HP can be attributed to organic nitrogen atom N (−3). In comparison, the E_b_ value of N (+5) in ammonium (NH_4_^+^) is about 401 eV. The E_b_ value of 169.0 eV was determined from the S*2p* band spectrum, which is typical for sulfur in sulfate ions [36]. The E_b_ value of 532.0 eV in the O*1s* band spectrum indicated the coexistence of inorganic oxygen (in SO_4_^2−^) and organic oxygen atom (in COO^−^ and C-O-C). Their E_b_ values were about 531.8 and 532.5 eV, respectively.

By comparison, the E_b_ value of O*1s* in COM-HP and COD-HP compounds increased, which appeared at 532.6 ± 0.1 eV. Meanwhile, the E_b_ value for Ca*2p* decreased by 1.1 eV compared with that of COM and COD crystals. The spin-orbit splitting of Ca*2p*_1/2_ and Ca*2p*_3/2_ are clearly observed at 346.1 ± 0.2 eV and 349.6 ± 0.2 eV, respectively, and their difference in value was about 3.5 eV.

**Table 1:** Binding energy (eV) of HP, COM, COD and their compounds.

Sample	C*1s*	N*1s*	S*2p*	O*1s*	Ca*2p*_1/2_	Ca*2p*_3/2_
COM	285.0			531.7	347.2	350.7
COD	285.0			531.9	347.4	350.9
HP powder	285.0	399.5	169.0	532.0		
COM-HP	285.0	400.3	169.2	532.5	346.0	349.5
COD-HP	285.0	400.1	169.3	532.7	346.3	349.8

The binding energy in XPS spectra depends on a small but measurable chemical shift due to the atomic charges localized both on the ionized and on the neighboring atoms. Therefore, it is related to net atomic charges. The factors that affect the measured XPS binding energy depend on the nature of the chemical surroundings, so a direct experimental characterization of such important quantities as the energy of molecular orbital levels and atomic charge distributions within the coordination sphere is available [37].

A displacement of E_b_ values greater than 0.2 eV indicates chemical bonding or dissociation. Our results showed that the E_b_ value of Ca*2p* decreased by 1.1 eV, whereas that of O*1s* increased by 0.6 eV (Table 1). It is clear that the lone pair of electrons from the oxygen atoms shifted into the metal atoms and formed a complexation. That is, HP was coordinated to the Ca^2+^ ions on the surface of nano COM and COD crystals through the O atom. The charge transfer from the O atoms of HP to the Ca^2+^ ions led to a decrease in charge density and an increase in the binding energy of O atoms.

According to [38] the E_b_ value of O*1s* increased by 3.0 eV after O atom was coordinated to Ca^2+^ ion. However, the E_b_ value in this study only increased by 0.6 eV because only part of the oxygen atoms of HP was coordinated with Ca^2+^ ions.

**Figure 7:**
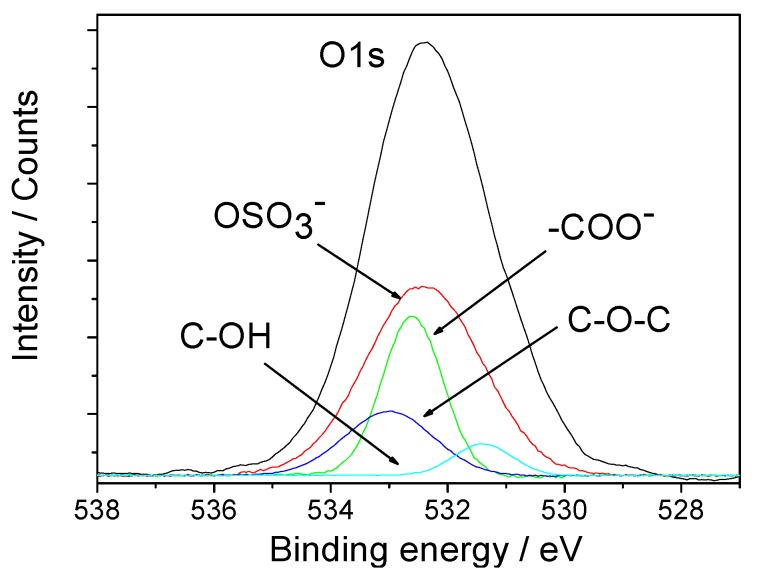
X-ray photoelectron spectra showing O*1s* core level of various oxygen atoms in COM-HP compounds.

The binding energy peak of O*1s* was resolved to differentiate the various interactions among the COO^−^, -OH, C-O-C, and OSO_3_^−^ groups and the calcium oxalate crystals. Figure 7 shows the O*1s* core level of various oxygen atoms in COM-HP compounds. The E_b_ value of the O*1s* photoelectron peaks of various oxygen atoms is listed in Table 2. The asymmetry of the O*1s* binding energy peak indicated the coexistence of several types of oxygen atoms. The respective O*1s* binding energy peaks were resolved by the computer software at 532.4, 532.6, 533.0, and 531.4 eV. Their intensity ratio was approximately 12:10:4:2, which corresponded to the different types of oxygen atoms in COM-HP compounds, namely, the oxygen atom peaks in the OSO_3_^−^, COO^−^, C-O-C, and C-OH groups, respectively [39,40,41]. This intensity ratio of the various oxygen atoms listed in Table 2 differed from that of the compound COM-HP, which may be attributed to the presence of O atoms in the COM crystals and the non-stoichiometric ratio between HP and COM. Compared with the previous coordination, the E_b_ value of O*1s* in the OSO_3_^−^ and COO^−^ groups had significantly increased, thereby indicating that the two kinds of O atoms in HP were coordinated to Ca^2+^ ions on the surface of COM.

**Table 2:** Core level binding energies of the O*1s* photoelectron peaks of various oxygen atoms in COM-HP compounds/eV.

Kind of O Atom	-OSO_3_^−^	-COO^−^	C-O-C	-C-OH
Number of O atoms	12	10	4	2
Before coordination	531.1 [39]	532.1 [40]	532.9 [41]	531.2 [40]
After coordination	532.4	532.6	533.0	531.4

### 2.5. Mechanism of Calcium Oxalate Stone Formation

**Figure 8:**
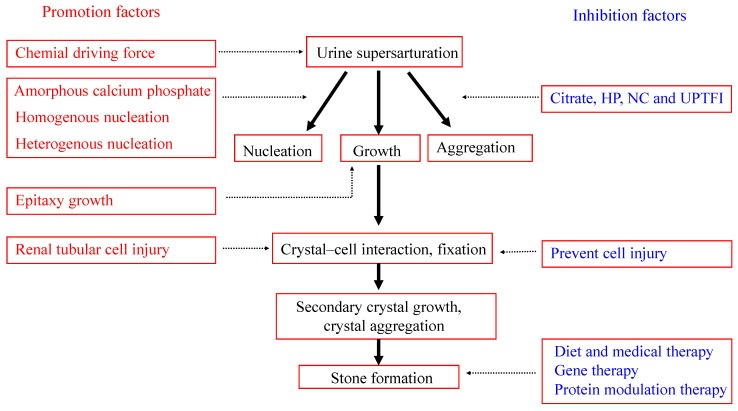
Schematic diagram for the formation and inhibition mechanism of calcium oxalate renal stone formation.

Figure 8 shows a schematic diagram for the growth and inhibition mechanism of calcium oxalate renal stone formation. The supersaturation of stone-forming salts in urine is the chemical driving force of urinary stone formation. A series of chemical thermodynamics reactions and kinetics processes occurs during urolithiasis, including crystal nucleation, growth, aggregation, dissolution, and solid phase transformation [42,43,44]. Patients with renal stones often excrete high concentrations of lithogenic materials, which are risk factors for stone formation. The increased excretion of stone-forming salts in urine could promote the formation of these types of stones, as well as the formation of other stone-forming salts by epitaxial growth. For example, calcium phosphate crystallization could promote the formation of CaOx stones.

The physiological importance of the calcium phosphate phase deserves more attention than calcium oxalate because dicalcium phosphate dihydrate and amorphous calcium phosphate could easily grow into large clusters of approximately 50 μm within a short period of time [4,24]. The fast-growing amorphous calcium phosphate was thought to serve initially as a “glue” to randomly consolidate 4–10 μm COM and dicalcium phosphate dihydrate crystallites. When conditions are kinetically favorable but thermodynamically unfavorable, the generating unstable phase would be successively converted into a stable phase.

Moreover, the process of renal tubular cell injury is of key importance during renal stone formation [45]. The development of medications that prevent renal tubular cell injury will provide a novel strategy for preventing this disease. Crystals easily become attached to injured renal tubular cells. The crystal and crystal aggregates grow, and finally a stone is formed.

The size, morphology, and composition of urinary crystallites may also affect the stone formation. The increased size, increased sharpness of corner angle, and the increased proportion of COM in the urinary crystallites all had important effects on the formation of kidney stones [28,46].

However, various types of urine inhibitors in urine can slow down, or even inhibit, the formation of renal stones. These inhibitors include macromolecule inhibitors, such as GAGs, UPTF1, and nephrocalcin, as well as small molecular inhibitors such as citrate, magnesium, and pyrophosphate.

A proper diet and medical therapy can be used in patients with severe disease to inhibit stone formation. Increased water intake and reduced salt consumption should be recommended for patients with a history of kidney stones [47]. Potassium citrate is a potentially effective medication for hypocitraturia and calcium oxalate stones. Gene or protein modulation therapy is another research direction for the inhibition of renal stones [48,49].

## 3. Experimental Section

### 3.1. Reagents and Apparatus

Heparin (HP) was purchased from Sigma Co. (St. Louis, MO, USA), and all other chemicals used for the experiment were of analytical grade. The samples were characterized by a D/max 2400 type X-ray powder diffractometer (Rigaku Corporation, Tokyo, Japan), a Zetasizer Nano ZS nanoparticle size and Zeta potential (Malvern Instruments Ltd., Malvern, UK), a TECNAI-10 transmission electron microscope at an accelerating voltage of 100 kV (Philips, Eindhoven, The Netherlands) and an ESCA Lab MKII photoelectron spectrometer (VG Scientific Ltd., London, UK).

### 3.2. Preparation of Nano COM and COD Crystals

*COM-50 nm*: In an ethanol–water mixture solvent (V_H2O_:V_ethanol_ = 1:1), 100 mmol/L CaCl_2_ and 100 mmol/L K_2_Ox solution were prepared. The two solutions with equal volume were rapidly mixed at 25 °C. The final concentration of the reactants was *c*(CaCl_2_) = *c*(Ox^2−^) = 50 mmol/L. After reacting for 6 min under high-speed stirring (1250 rpm), the product was centrifugally separated and then ultrasonically washed twice with anhydrous ethanol. After it was dried, COM with a size of 50 ± 15 nm was obtained.

*COD-50 nm*: Up to 12 mL CaCl_2_ solution (4 mol/L) was added into buffer solution (188 mL, pH = 6.8) at 5 °C. Then, K_2_Ox solution (50 mL, 250 mmol/L) was directly poured into it under rapid stirring (1250 rpm) at constant temperature of 5 °C. After reacting for 5 min at the same stirring speed, the product was centrifugally separated and then ultrasonically washed twice with anhydrous ethanol. After it was dried, COD with a size of 50 ± 15 nm was obtained. XRD measurements confirmed that the obtained products were COM and COD crystals.

### 3.3. Effect of HP on Aggregation, Zeta Potential, and Phase Change of Nano COM and Nano COD Crystals

After 5.0 mg COM or 5.5 mg COD was added to solution of *c*(HP) (25 mL, 0, 0.010, 0.020, 0.050, 0.10, 0.20, 0.50, 1.0 and 2.0 mg/L), respectively, the suspensions of nano COM or nano COD crystals with a concentration of approximately 1.6 mmol·L^−1^ in 0.15 mmol·L^−^^1^ NaCl were formed after ultrasonically dispersion for 5 min. After the suspensions were stored at 37 °C for 0, 6, 12, 24 and 48 h, respectively, the particle size and Zeta potential of the crystals were detected at 37 °C by nanoparticle size Zeta potential analyzer [50]. All the data were the average values of three parallel tests. The components of crystals were detected by XRD. Approximately 100 μL of the suspensions of nano COM or nano COD crystals was placed on clean glass slides by using a microsyringe. The glass slides were dried in a vacuum desiccator for 1 d to make samples that could be used for XRD characterization.

### 3.4. Measurement of Nano Crystals by High-Resolution Transmission Electron Microscopy (HRTEM)

After the suspension of nano COM or nano COD crystals was subjected to ultrasound treatment for 5 min, approximately 5 μL of the suspension was submerged in a copper mesh by a microsyringe, The mesh was stored in a desiccator for 2 d prior to HRTEM analyses.

### 3.5. X-ray Photoelectron Spectroscopy

XPS spectra were used to comparatively study nano COM, nano COD, HP and their compounds (COM-HP and COD-HP) formed by interaction between 2.0 mg/L HP and COM, COD crystals for 48 h respectively.

A two-step procedure was used in these studies. At first wide scanning spectra of the HP, COM-HP and COD-HP were recorded. Careful inspection of the spectra allows us to assign the observed peaks to particular components. The wide scans were conducted from 0 to 1200 eV with a pass energy of 100 eV. In the second step the most characteristic peaks were recorded in narrow ranges of binding energy in order to obtain a better statistic and resolution required for an optimal spectral manipulation. The elements, C, N, S, O, and Ca, were scanned over the energy range of 282–294, 395–402, 160–185, 528–540, and 344–354 eV, respectively, with the pass energy of 20 eV. The charging shift was corrected with the C*1s* line emitted from neutral hydrocarbon.

## 4. Conclusions

The polyanionic HP molecule contains -OSO_3_^−^, -COO^−^, and -OH groups. It could not only form coordination bonds with Ca^2+^ ions on the surface of calcium oxalate crystals but also hydrogen bonds with Ox^2−^ ions on the crystal surface. XPS confirmed the coordination of -OSO_3_^−^ and -COO^−^ groups with the Ca^2+^ ions of COM and COD crystals. After HP was adsorbed on the surface of nano CaOx (including COM and COD) crystals, the negative charges on the crystallite surface increased, which made the Zeta potentials negative. Thus, the electrostatic repulsion force between the crystals increased and the aggregation degree of crystals reduced. Consequently, the stability of nano COM or COD suspension improved. These results are conducive to inhibition of the formation of CaOx stones.
